# Control of Reactive Oxygen Species for the Prevention of Parkinson’s Disease: The Possible Application of Flavonoids

**DOI:** 10.3390/antiox9070583

**Published:** 2020-07-03

**Authors:** Tae Yeon Kim, Eunju Leem, Jae Man Lee, Sang Ryong Kim

**Affiliations:** 1 School of Life Sciences, BK21 plus KNU Creative BioResearch Group, Kyungpook National University, Daegu 41566, Korea; taetaey@hanmail.net (T.Y.K.); ejlll1005@knu.ac.kr (E.L.); 2 Department of Biochemistry and Cell Biology, Cell and Matrix Research Institute, BK21 Plus KNU Biomedical Convergence Program, School of Medicine, Kyungpook National University, Daegu 41944, Korea; jaemanlee@knu.ac.kr; 3 Institute of Life Science & Biotechnology, Kyungpook National University, Daegu 41566, Korea; 4 Brain Science and Engineering Institute, Kyungpook National University, Daegu 41566, Korea

**Keywords:** Parkinson’s disease, reactive oxygen species, flavonoid, neuroprotection, neuro-inflammation

## Abstract

Oxidative stress reflects an imbalance between the production of reactive oxygen species (ROS) and antioxidant defense systems, and it can be associated with the pathogenesis and progression of neurodegenerative diseases such as multiple sclerosis, stroke, and Parkinson’s disease (PD). The application of antioxidants, which can defend against oxidative stress, is able to detoxify the reactive intermediates and prevent neurodegeneration resulting from excessive ROS production. There are many reports showing that numerous flavonoids, a large group of natural phenolic compounds, can act as antioxidants and the application of flavonoids has beneficial effects in the adult brain. For instance, it is well known that the long-term consumption of the green tea-derived flavonoids catechin and epigallocatechin gallate (EGCG) can attenuate the onset of PD. Also, flavonoids such as ampelopsin and pinocembrin can inhibit mitochondrial dysfunction and neuronal death through the regulation of gene expression of the nuclear factor erythroid 2-related factor 2 (Nrf2) pathway. Additionally, it is well established that many flavonoids exhibit anti-apoptosis and anti-inflammatory effects through cellular signaling pathways, such as those involving (ERK), glycogen synthase kinase-3β (GSK-3β), and (Akt), resulting in neuroprotection. In this review article, we have described the oxidative stress involved in PD and explained the therapeutic potential of flavonoids to protect the nigrostriatal DA system, which may be useful to prevent PD.

## 1. Introduction

Parkinson’s disease (PD) is a chronic and slow progressive neurological disease that is associated with the progressive degeneration of dopaminergic (DA) neurons in the substantia nigra (SN) and reduced levels of striatal dopamine and its metabolites in the adult brain [1,2,3]. Affected patients experience motor function impairments including tremors, rigidity, and bradykinesia, along with other symptoms such as dementia, depression, insomnia, and anosmia [1,2,3,4]. The pathogenic mechanisms of PD causing the degeneration of the nigrostriatal DA system still remain unclear, however, it is well known that the upregulation of risk factors is involved in the pathogenesis and progression of PD [4,5,6,7], and the following circumstances are highly responsible for the onset of PD: neuroinflammation [8,9], mismanagement of apoptosis [10,11,12] and autophagy [10,11,12,13], genetic mutations [5,14], neurotrophic support failure [6,8,15,16,17], and oxidative stress [5,18,19]. In particular, oxidative stress is considered to be a key risk factor of PD due to the vulnerability of DA neurons to oxidative stress caused by the excessive production of reactive oxygen species (ROS; free radicals) [14,20,21], resulting from the dopamine metabolism [22,23], mitochondrial dysfunction [14,19,24], neuroinflammation [3,8,9,25], and iron accumulation [26,27] in the SN.

Flavonoids are an enormous class of natural products derived from the plant kingdom composed of a variety of low molecular weight polyphenolic compounds; more than 8000 varieties of flavonoids have currently been identified [28,29,30,31,32,33,34]. The term “flavonoid” originates from the Latin word “flavus,” meaning yellow, but flavonoids exist in a range of colors, including white, pale yellow, yellow, orange, scarlet, red, purple, and blue [29,30,33,35,36,37]. Consequently, the biological feature of flavonoids as pigments is responsible for the color of fruits, leaves, and petals, and flavonoids are also responsible for the scent of flowers and fruits [37]. These visual and olfactory characteristics play an important role in pollination and the dispersion of seeds and spores by attracting insects and animals [36,37,38]. Consequently, flavonoids are ubiquitously found in flowers, fruits, herbs, vegetables, nuts, grains, plant-derived beverages, and even chocolate [29,30,35,36,39]. Within the cell, they are present in chloroplasts, a type of plastid that is the cellular organelle responsible for photosynthesis. Photosynthesis, observed in plants, cyanobacteria, and algae, is an anabolic metabolism that produces glucose by converting light energy to chemical energy through electron transfer induced by the reduction of carbon dioxide (CO_2_) and the oxidation of water [30,33,36,37,38,40]. Redox reactions, which occur during photosynthesis, are involved in triggering harmful oxidative stress. Because flavonoids are abundant in cellular organelles, we can infer that they mediate ROS scavenging mechanisms to protect plants against the oxidative stress that results from anabolic metabolism [30,33,36,37,38,40] and contribute to the maintenance of redox balance because of their thermodynamically low electron potentials [33,36,41]. Additionally, many flavonoids can provide health-promoting effects through various cellular signaling pathways associated with cell proliferation and survival [42,43,44]. In this review, we describe the role of ROS as it relates to the mismanagement of oxidative stress in PD and explain the therapeutic potential of flavonoids as neuroprotective agents against PD.

## 2. The Role of Oxidative Stress in Parkinson’s Disease

Comprehensive studies on hereditary and sporadic PD suggest that loss of DA neurons in the adult brain can be induced by various neurotoxic events, such as ROS production [14,24,45], mitochondrial dysfunction [4,19], protein aggregation [11,13], aberrant apoptosis signaling pathways [10,12], downregulation of neurotrophic factors [6,15,16,17,46], and excessive inflammation [3,8,9]; these pathogenic events work together to degenerate DA neurons. Although the fundamental etiology of PD remains unclear, it has been ascertained that the oxidative stress induced by excessive ROS production is widely involved in the pathogenesis of PD [5,18].

Oxidative stress is characterized as the imbalance between oxidants, which produce ROS and antioxidants, which remove free radicals. In healthy conditions, the levels of oxidants and antioxidants maintain balance [5,45]. However, excessive ROS production or a deficiency of antioxidants generates oxidative stress, which damaging biomolecules (DNA, proteins, lipids, etc.) that lead to neurodegenerative diseases. Oxidative stress leads to cellular dysfunction and demise, especially playing a major role in the degeneration of DA neurons in the pathogenesis of Parkinson’s disease [47]. Consequently, preventing ROS production and reducing oxidative stress may be a crucial therapeutic target for PD treatment. We will further discuss the sources that generates ROS, how various types of ROS are produced, and the biological effects of ROS.

### 2.1. ROS Generating Sources

Although the brain represents only 2% of the whole body weight, it requires about 20% of the oxygen consumed by the whole body, making it susceptible to ROS. Besides this main factor, there are various factors affecting the brain’s sensitivity to ROS. Abundant redox-active metals (iron, copper, etc.) existing in the brain play a role in catalyzing ROS, and any deficiency in the antioxidant defense system that reduces ROS levels makes the brain more vulnerable to ROS. A high level of polyunsaturated fatty acids (PUFAs) in the cell membrane also affects the brain [48]. Additionally, among neurons in various brain regions, DA neurons in substantia nigra *pars compacta* (SNpc) are highly vulnerable to oxidative stress [14,20,21]. By ROS production, various neurodegenerative diseases, such as PD, Alzheimer’s disease (AD), Huntington’s disease (HD), multiple sclerosis (MS), and amyotrophic lateral sclerosis (ALS), are induced by biochemical alterations [49]. We will further discuss herein the major sources of ROS, including mitochondrial dysfunction, dopamine metabolism, neuroinflammation, iron accumulation, and deficiency of antioxidant defense.

#### 2.1.1. Mitochondrial Dysfunction

Mitochondria generate energy for cellular metabolism by the oxidative phosphorylation (OXPHOS) system. Oxidative phosphorylation takes place through the electron transport chain (ETC), which consists of four protein complexes (complex I, II, III, IV) and chemiosmosis known as adenosine triphosphate (ATP) synthase, which is located in the inner mitochondrial membrane [50]. The electron transport chain is a series of electron transporters in the mitochondria that transfer electrons *via* redox reactions. The electrons from NADH (the oxidized form of nicotinamide adenine dinucleotide) and FADH_2_ (the hydroquinone form of flavin adenine dinucleotide) pass through electron transport chain complexes and transfer to molecular oxygen reducing it to form water. Additionally, chemiosmosis pumps protons into the mitochondrial matrix, from which they are pumped out to the intermembrane space by electron transport chain complexes, generating ATP. When NADH approaches complex I it becomes NAD^+^ (the reduced form of nicotinamide adenine dinucleotide) by transferring its electrons and protons to complex I. As a result, complex I becomes supercharged. Like NADH, FADH_2_ also approaches complex II, transferring its electrons to complex II and becoming FADH (semiquinone; the reduced form of FADH_2_) [51]. However, complex II is not supercharged and does not pump protons out into the intermembrane space. The electrons remaining in complex I and complex II move to coenzyme Q (CoQ), which transfers its electrons sequentially to complex III, cytochrome C, and complex IV. Then the electrons are transferred to oxygen, the final electron acceptor, and form water (H_2_O) molecules. Supercharged complex I, complex III, and complex IV acquire the energy to pump the protons from the mitochondrial matrix to the intermembrane space producing a number of protons in the intermembrane space. ATP synthase uses this proton to turn adenosine diphosphate (ADP) into massive amounts of ATP, which is a high energy molecule that provides energy to various life-sustaining activities in living cells, including neurons’ aerobic respiration [52]. This process is called oxidative phosphorylation and in this oxidative phosphorylation system, electron transport chain complex I and complex III are the main producers of ROS, including hydrogen peroxide and superoxide anion, and this production is enhanced when the electron transfer is reduced by the increased membrane potential [53]. By the electron leakage, the oxygen interacts with unpaired electrons induced by nicotinamide adenine dinucleotide phosphate oxidase (NADPH oxidase) at complex I and generates superoxide anion. Subsequently, superoxide anion (O_2_^•−^) forms hydrogen peroxide (H_2_O_2_) by mitochondrial superoxide dismutase (SOD) and this ROS is released to the cellular cytosol and nucleus, generating oxidative stress. Hydrogen peroxide is converted to hydroxyl radical (^−^OH) by the Fenton reaction, which leads to more oxidative stress [54]. With pathological conditions, mitochondrial dysfunction can cause excessive ROS production [55]. The reduction in complex I activity has been demonstrated in the SN of PD patients [56]. Additionally, complex I inhibitors, such as 1-methyl-4-phenyl-1,2,3,6-tetrahydropyridine (MPTP), rotenone, and paraquat, cause DA neuronal loss by increasing ROS generation. 1-methyl-4-phenylpyridinium (MPP^+^), a metabolite of MPTP, is a neurotoxin that inhibits complex I leading to the blockage of electron translocation through electron transport chain. These results suggest that a depletion of ATP and ROS generation cause Parkinsonism [57]. Post-mortem studies also showed a decrease in complex I in the brain of PD patients [58,59].

#### 2.1.2. Dopamine Metabolism

It has been demonstrated that oxidative stress is involved in the degeneration of DA neurons in PD. Dopamine is a representative neurotransmitter in DA neurons, which plays a major role in motor activity. Dopamine is synthesized by the following mechanism; L-3,4-dihydroxyphenylalanine (L-DOPA), the precursor of dopamine, is synthesized from the amino acid tyrosine by tyrosine hydroxylase (TH), and L-DOPA synthesizes dopamine using DOPA decarboxylase (DDC). The monoaminergic synaptic vesicles uptake dopamine by vesicular monoamine transporter 2 (VMAT2) and a complex consisting of tyrosine hydroxylase, DOPA decarboxylase, and VMAT2 is formed, which prevents the release of dopamine to the cytosol where dopamine oxidation is activated [60]. Thus, reducing VMAT2 expression leads to dopamine toxicity and DA neuron loss, which generates progressive nigrostriatal neurodegeneration [61]. Although the TH, DOPA decarboxylase, and VMAT2 complex prevents the release of dopamine to the cytosol and normally stores dopamine in synaptic vesicles, damaged neurons with impaired dopamine reuptake release an excessive amount of cytosolic dopamine outside the synaptic vesicle and activates dopamine oxidation by enzymatic metabolism or by auto-oxidation [18]. Dopamine oxidation by enzymatic metabolism is a process in which dopamine is degraded into its metabolites, such as 3,4-dihydroxyphenlacetic acid (DOPAC) by monoamine oxidase (MAO) or 3-methoxytyramine (3-MT) by catechol-O-methyl transferase (COMT), generating a final metabolic product known as homovanillic acid (HVA). Although dopamine itself is not toxic, dopamine metabolites are damaging to DA neurons by MAO producing H_2_O_2_, which is one of the representative ROS. Dopamine oxidation is also generated by the auto-oxidation of dopamine [62]. During dopamine oxidation the two electrons and two protons in two hydroxyl groups of dopamine transfer to oxygen, which reduces to superoxide radical and generates dopamine-quinone (DAQ), which is a highly reactive oxidized dopamine. By oxidation, not only the generation of ROS, including hydrogen peroxide, superoxide anion, and hydroxyl radicals, but also the generation of DAQ, which plays a crucial role in dopamine-related toxicity to DA neurons by modifying genes associated with PD, such as α*-synuclein* (α*-syn*), *parkin*, *DJ-1*, and *ubiquitin C-terminal hydrolase L1* (*UCH-L1*), degenerate DA neurons. Modification of PD-related genes lead to oxidative stress and inhibit the ubiquitin proteasome system (UPS), which regulate protein clearance. This leads to protein misfolding, inactivation, and aggregation [63]. Dopamine transporter (DAT) inactivation, mitochondrial dysfunction and impairment also occur, which is toxic to DA neurons implicated in the neurodegenerative process of PD [57]. Furthermore, DAQ is oxidized to aminochrome, which leads to the generation of the superoxide radical and the degradation of cellular NADPH oxidase, consequently forming the neuromelanin, the final product of dopamine oxidation, accumulated in the SNpc of the human brain [55].

#### 2.1.3. Neuroinflammation

Neuroinflammation is a protective mechanism of a nerve or of the central nervous system (CNS) against infection, toxic metabolites, autoimmunity, and traumatic brain injury to eliminate and destroy detrimental agents and injured tissues. This is characterized by the activation of glial cells mainly known as microglia and astrocytes in the brain [64]. Microglia are the resident innate immune cells in the CNS, which are responsible for immune defense for overall brain maintenance. These cells not only act as a neuroprotective cell by scavenging endogenous and exogenous substances, but also protect themselves from toxic levels of ROS [20]. In the normal condition of the healthy brain, inactive microglia are in the resting state, which maintain morphology of small cell bodies with ramified processes. However, in the neuropathological conditions, microglia are activated with the morphology changes of large cell bodies with short or no processes. Activated microglia also produce inflammatory mediators, such as inducible nitric oxide synthase (iNOS), cyclooxygenase-2 (COX-2), and pro-inflammatory cytokines, including tumor necrosis factor-α (TNF-α), interleukin-1 β (IL-1β), interleukin-2 (IL-2), interleukin-6 (IL-6), and interferon γ (IFN-γ), which are neurotoxic in the brain [65]. In neuroinflammation, activated microglia continuously release pro-inflammatory cytokines, and consequently induce chronic inflammation, which produces cytotoxic molecules, such as ROS and reactive nitrogen species (RNS) [66,67]. Since the midbrain contains more microglia than other brain regions, DA neurons would be more vulnerable to microglia leading to progressive DA neuron loss in PD by oxidative stress [68]. Post-mortem studies have shown an increase in activated microglia and pro-inflammatory cytokines, such as TNF-α, IL-1β, IL-6, and IFN-γ, which induces neuroinflammation in the brain of Parkinson’s disease patients [69,70]. Therefore, reducing high levels of ROS by controlling microglial activation may be an essential way to prevent the degeneration of the DA neurons in PD.

### 2.2. Production of ROS

Oxygen is an essential molecule regulating cellular activities, including energy metabolism, breathing processes, cell signaling, gene transcription, and homeostasis for all of the living organisms. While oxygen has beneficial roles in our life, forms of altered oxygen molecules known as ROS contribute to detrimental effects on our cells [49]. ROS is a group of highly reactive unstable molecules that originates from oxygen. ROS has beneficial effects in the normal condition with balanced production of oxidants and antioxidants; attacking various pathogens, having destructive effects on tumor cells, regulating cell signaling, activation of gene expression, immune responses, blood pressure modulation, smooth muscle relaxation, etc. [71]. However, the detrimental effects of ROS are more significant according to excessive ROS production.

ROS are classified as oxygen-centered radicals and oxygen-centered non-radicals depending on the existence of a free radical which contains one or two unpaired electrons. Oxygen-centered radicals include superoxide anion (O_2_^•−^), hydroxyl radicals (^•^OH), alkoxyl radicals (RO^•^), peroxyl radicals (ROO^•^), and hydroperoxyl radicals (HO_2_^•^). The oxygen-centered non-radicals include hydrogen peroxide (H_2_O_2_), hypochlorous acid (OCl^−^), and singlet oxygen (O_2_) (Table 1) [72,73].

Superoxide anion is the initial free radical of the oxygen-forming ROS. One electron from oxygen is transferred by oxidases, such as NADPH oxidase, COX and xanthine oxidase (XO) [74]. NADPH oxidation transfers the NADPH electron to oxygen in the mitochondrial electron transport chain, including mitochondrial complex I and complex III, resulting in the reduction of oxygen to produce superoxide anion, which is highly reactive:

NADPH → H^+^ + NADP^+^ (1)

O_2_ + e^−^ → ^•^O_2_^−^ (2)

Also, superoxide anions (O_2_^•−^) are prone to penetrate the mitochondrial membrane where hydrogen peroxide (H_2_O_2_) is produced by a dismutation reaction:

^•^O_2_^−^ + ^•^O_2_^−^ + 2H^+^ → H_2_O_2_ + O_2_ (3)

By SOD, superoxide anion is reduced to hydrogen peroxide, which is the least reactive form of ROS [75,76]. Superoxide dismutase is an important antioxidant enzyme to almost all the cells exposed to oxygen by inactivating superoxide anion to a more stable ROS form [77,78]. In addition to the production of hydrogen peroxide in the mitochondria, it can also be produced by peroxisomes, which contain peroxisomal enzymes, such as catalase. With metals involved in the Fenton reaction, hydrogen peroxide is also reduced to form hydroxyl radicals, which is the highly reactive, most harmful ROS [57,79]. Several in vivo and in vitro studies show that hydroxyl radicals are mediators of tissue damage also attacking dopamine [80,81]:

Fe_2_^+^ + H_2_O_2_ → Fe_3_^+^ + ^•^OH + OH^−^ (4)

In brief, subsequent processes of oxygen reduction induce ROS production, including superoxide anion, hydrogen peroxide, and hydroxyl radicals.

### 2.3. Biological Effects of ROS

Excessive ROS production or deficiency of antioxidants generate oxidative stress, which cause damage to biomolecules (DNA, proteins, lipids, etc.) in neuronal cells and the brain. High oxygen demand, which requires about 20% of the whole body, abundant redox-active metals (iron, copper, etc.) existing in the brain, deficiency of antioxidant defense system, and high levels of polyunsaturated lipids in the cell membrane make the brain vulnerable to ROS. In common with the brain, but irrelevantly, the neuronal membrane is also vulnerable to ROS with abundant polyunsaturated fatty acids [48,49]. Biomolecular components, which are susceptible to free radicals (ROS/RNS), experience biochemical modification. This causes modification of cell function and cell death inducing neurodegenerative diseases. We will discuss the biological effects of the ROS typically including DNA/RNA oxidation, lipid oxidation, and protein oxidation.

#### 2.3.1. DNA/RNA Oxidation

DNA is a double-stranded molecule made up of nucleotides, which contains deoxyribose sugar, phosphate groups, and nucleotide bases, such as adenine (A), guanine (G), cytosine (C), and thymine (T). Interaction with oxygen atoms induces oxidation of DNA bases, which damage DNA. Among DNA bases, guanine is the most easily oxidized DNA base, since guanine has the lowest reduction potential of the four DNA bases [82]. Guanine interacts with hydroxyl radicals and generates one of the major products of DNA oxidation, such as 8-oxo-2′-deoxyguanosine (8-oxo-dG), among more than 20 oxidative DNA base products that have been identified [83]. By producing 8-oxo-dG, guanine binds with adenine instead of cytosine inducing G:C to T: A transversion mutations, which modifies DNA [84]. DNA oxidation cause modification of cell function and cell death, and the DNA repair system is not normally activated. It has also been reported that 8-oxo-2′-deoxyguanosine levels in DNA increase in SN of patients with PD [85]. Additionally, RNA oxidation is similar to DNA oxidation. Since RNA is located close to ROS sites in the cell it is more easily oxidized than DNA resulting in ribosomal dysfunction and nucleotide breakage [74].

#### 2.3.2. Lipid Oxidation

In the cell membrane there are high levels of PUFAs in the phospholipid bilayers, which makes the brain more vulnerable to ROS [53]. ROS, such as hydroxyl radicals, peroxyl radicals, and alkoxyl radicals, take electrons from the PUFAs in the cell membranes easily, and produce water and fatty acid radicals (L). This unstable fatty acid radical interacts with oxygen, which produces unstable fatty acid peroxyl radical (LOO) and this radical also interacts with another PUFA producing another fatty acid radical and a lipid hydroperoxide (LOOH) [86,87]. Repeated cycles of this lipid oxidative degradation process known as lipid peroxidation can affect serious damage to the cell membranes resulting in various human diseases, such as neurodegenerative disorders, cancer, diabetes, and atherosclerosis [88]. While the levels of PUFAs are reduced in the brain of Parkinson’s disease, increased levels of malondialdehyde and hydroperoxides, which are the final products of lipid peroxidation of PUFAs, have been shown in the SN of PD patients [89,90]. Additionally, lipid peroxidation induced by the misfolding of α-Syn has been shown to play an important role in neuronal cell loss in PD. Neurons were protected from cell death induced by misfolding of α-Syn by inhibition of lipid peroxidation [91].

#### 2.3.3. Protein Oxidation

ROS can damage proteins by oxidation of amino acids, because all amino acids are susceptible to oxidation, especially cysteine and methionine [92,93]. Protein oxidation is initiated by interactions with hydroxyl radicals inducing protein modifications, such as protein-protein cross-linking, amino acid side chain modification, protein fragmentation by oxidation of the protein backbone, and enzyme inactivation resulting in activity loss. There are some examples of modification of proteins, such as oxidization of methionine converting to methionine sulfoxide; histidine to 2-oxohistidine, asparagine, and aspartic acid residues; tryptophan to formylkynurenine and kynurenine; phenylalanine and tyrosine to a hydroxy derivative [94,95]. Also, most of the oxidative proteins must be eliminated by proteolytic degradation, however the decrease of the efficiency of proteolysis will increase the oxidatively modified proteins, which disrupts cellular function leading to a number of diseases, including PD, AD, diabetes, and ALS [28,96].

## 3. Inhibition of Oxidative Stress by Antioxidants: Application as a Therapeutic Strategy against PD

To inhibit the oxidative stress for therapeutic strategies against PD, removing ROS by utilizing antioxidants is an efficient way to protect cells from cell death. The antioxidant system plays a wide range of roles by blocking the secondary production of toxic metabolites, blocking inflammatory mediators, blocking ROS production, converting high toxic ROS to a less toxic ROS, eliminating ROS, repairing the damaged biomolecular induced by ROS, enhancing endogenous antioxidant defense system, etc. To protect cells from oxidative stress these antioxidant systems act cooperatively [97,98]. The antioxidant system can be classified into two main groups: enzymatic antioxidants and non-enzymatic antioxidants. The enzymatic antioxidants include SOD, catalase, and glutathione peroxidase (GPx), which act as primary defense against ROS. Additionally, non-enzymatic antioxidants include glutathione (GSH), selenium, carotenoids, flavonoids, vitamin C (ascorbic acid), and vitamin E (α-tocopherol), which act as secondary defense against ROS (Table 2) [99].

### 3.1. Enzymatic Antioxidants

Enzymatic antioxidants include SOD, catalase, and GPx, which act as primary defense against ROS inhibiting oxidative stress. While SOD decreases the level of superoxide anion [100], catalase and GPx decrease the level of hydrogen peroxide [101].

#### 3.1.1. Superoxide Dismutase (SOD)

The SOD reduces superoxide anion to hydrogen peroxide and oxygen, which converts highly toxic ROS (superoxide anion) to a less toxic ROS (hydrogen peroxide) [75,76]. This antioxidant is an important antioxidant enzyme to almost all the cells exposed to oxygen by inactivating superoxide anion to a more stable form of ROS. Since superoxide dismutase contains metal ions, such as copper (Cu), zinc (Zn), iron (Fe), or manganese (Mn), this enzyme is classified into three types generally depending on the protein fold and the metal cofactor. The Cu/Zn type enzymes bind to Cu and Zn, Fe and Mn type enzymes bind to Fe or Mn, and nickel (Ni) type enzymes bind to Ni. In the human body, SODs are also classified into three isoforms: SOD1, SOD2, and SOD3. SOD1 is a cytosolic Cu/Zn-SOD (Cu/Zn type) located in the cytosol and mitochondrial intermembrane space, which removes cytosolic superoxide anions. SOD2 is a mitochondrial manganese-SOD (MnSOD) located in the mitochondrial matrix, which eliminates mitochondrial superoxide anions. SOD3 is extracellular SOD (Cu/Zn type), which is located in extracellular fluids [71,102]. Several studies indicated the importance of SODs by deficiency of SOD in genetically engineered mice [103].

#### 3.1.2. Catalase

Catalase is an enzyme produced from peroxisomes and this antioxidant protects cells from oxidative stress induced by hydrogen peroxide. This enzyme is a hemoprotein composed of a tetramer of four polypeptide chains and contains four porphyrin heme groups, which make it prone to react with the hydrogen peroxide [97,104]. In the normal condition of the peroxisome, catalase is activated to change hydrogen peroxide into water and oxygen by the dismutation reaction using Mn or Fe as a cofactor [71]:

H_2_O_2_ + H_2_O_2_ → 2H_2_O + O_2_ (5)

This reaction blocks the accumulation of hydrogen peroxide and reduces the ROS level. Damaged peroxisome however down-regulates catalase and hydrogen peroxide is released to the cytosol resulting in oxidative stress [20].

#### 3.1.3. Glutathione Peroxidase (GPx)

GPx is another enzyme that reduces ROS levels induced by hydrogen peroxide. Hydrogen peroxide is removed by interacting with reduced GSH, which is a reductant that converts to GSSG (oxidized form of GSH) by GPx [105]:

H_2_O_2_ + 2GSH → GSSG + 2H_2_O (6)

In mammals, there are eight isoforms of GPx, five selenium-containing glutathione peroxidases (GPx1, GPx2, GPx3, GPx4, and GPx6) and three non-cysteine glutathione peroxidases (GPx5, GPx7, and GPx8). These isoforms also function as antioxidants at different locations in the cells. GPx1 is expressed in the mitochondria and cytosol, GPx2 in the intestinal epithelium, GPx3 in the plasma, GPx5 in the epididymis, and GPx6 in the olfactory epithelium. GPx4 has a function to protect membranes from oxidative stress and GPx7, GPx8 have low glutathione peroxidase activity [106].

### 3.2. Non-enzymatic Antioxidants

Non-enzymatic antioxidants contain metabolic antioxidants and nutrient antioxidants. Metabolic antioxidants, such as GSH, transferrin, and lipoic acid, are generated by metabolic reactions in the cell. Nutrient antioxidants involving exogenous antioxidants, such as vitamin C, vitamin E, cysteine, β-carotene, and flavonoids, are consumed through food or food supplements [71].

#### 3.2.1. Glutathione (GSH)

GSH is an antioxidant consisting of tripeptide synthesized from glutamate, glycine, and cysteine and has a protective function against ROS [56]. ROS, such as hydrogen peroxide, hydroxyl radicals, and superoxide anion, are removed non-enzymatically by interacting with the reduced form of GSH. Also, by enzymatic reactions, GSH remains in an oxidized form or a reduced form. In the GPx reaction, GSH is the electron donor that removes hydrogen peroxide enzymatically and is converted to oxidized form of GSH (GSSG):

H_2_O_2_ + 2GSH → GSSG + 2H_2_O (7)

By the GSH reductase reaction, the oxidized form of GSH regenerates the reduced form of GSH utilizing NADPH [107]:

GSSG + NADPH + H^+^ → 2GSH + NADP^+^ (8)

Researchers found that the level of reduced GSH in the SN in PD is decreased, resulting in DA neuronal loss induced by ROS formation and lipid peroxidation [108]. So, GSH levels are an important factor in controlling ROS production and neurodegeneration.

#### 3.2.2. Coenzyme Q (CoQ)

CoQ is an antioxidant enzyme cofactor involved in the mitochondrial electron transport chain, which transfers electrons in complex I and complex II to complex III. By increasing complex I activity, CoQ decreased production of hydrogen peroxide in PD, which reduce the levels of ROS [109]. It is reported that the levels of CoQ in patients with PD is decreased along with reduced complex I activity compared with the control groups [110,111]. These beneficial effects of CoQ related with neuroprotection have been confirmed through following studies that the oral administration of CoQ decreased mitochondrial dysfunction by increasing brain mitochondrial concentration in PD animal models, and also suppressed the loss of striatal dopamine and DA axons induced by MPTP toxicity [112]. These results indicate that CoQ may be a useful antioxidant of PD.

#### 3.2.3. Vitamin C, E

Vitamin C and vitamin E are the main exogenous antioxidants, which attenuates effects of ROS and inhibits lipid peroxidation of the cell membrane. Vitamin C also known as ascorbic acid is a water-soluble antioxidant, which must be supplied through food since it cannot be synthesized in the human body. It plays a significant role of scavenging ROS, such as hydrogen peroxide, hydroxyl radicals, and superoxide anion, and inhibiting lipid peroxidation of the cell membrane. Also, it is a cofactor for antioxidant enzymes participating in attacking ROS [53]. Several studies have demonstrated that vitamin C is a neuroprotective antioxidant in a variety of models of neurodegenerative diseases and some studies showed that vitamin C can prevent dopamine-mediated toxicity [113].

Vitamin E is a lipid-soluble antioxidant, which is a necessary nutrient that needs to be supplied through food. It is responsible for chain-breaking and the preservation of the cell membrane damage against ROS, such as peroxyl radicals, by blocking lipid peroxidation of the cell membrane [74]. There are studies of PD animal models showing that vitamin E has a neuroprotective effect against ROS induced by 6-hydroxydopamine (6-OHDA), a neurotoxin that destroy DA and noradrenergic neurons in the brain [114].

However, vitamins may not be an effective antioxidant for therapeutic usage according to some other studies. Some studies showed that vitamins did not significantly affect mortality and other studies also showed significant increase in mortality by administration of a single vitamin or a combination of other antioxidants with vitamins [115]. We will further discuss more about flavonoids, which are some of the main non-enzymatic antioxidants.

## 4. Natural Antioxidants: Flavonoids

**Figure 1:**
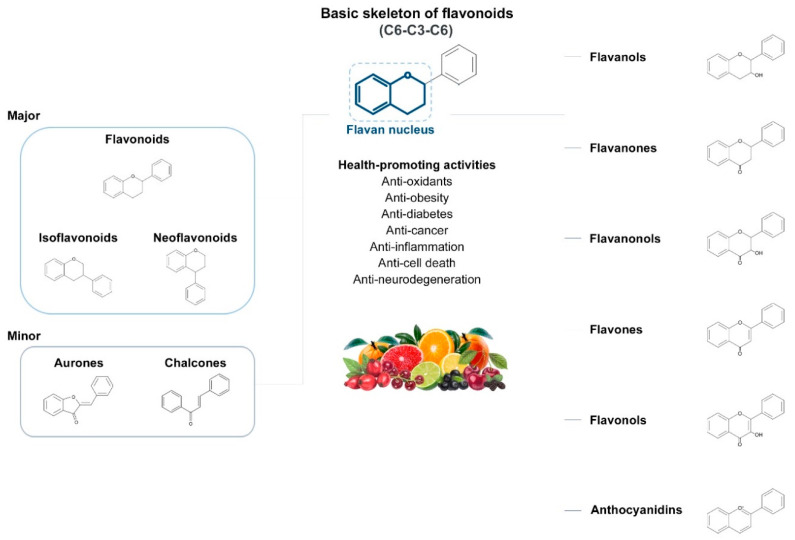
Classification of flavonoids based on chemical structure.

Flavonoids, a group of plant secondary metabolites, have a polyphenolic structure characterized by a flavan skeleton (also known as a flavan nucleus) consisting of 15 carbons, which are arranged as C_6_-C_3_-C_6_ (Figure 1) [28,29,30,31,32,33,34]. Flavan is the simplest structure in the flavan class and consists of three phenolic rings based on a phenyl-substituted chromane (C_9_H_6_O) structure, a heterocyclic chemical compound (Figure 1) [29,36]. Flavonoids are categorized depending on where the phenyl is substituted on the chromane: the general form of flavonoids is substituted with phenyl at C2 on the chromane (2-phenylbenzopyran), isoflavonoids (3-phenylbenzopyran), and neoflavonoids (4-phenylbenzopyran) possess the substituted form of chromane with phenyls at C3 and C4, respectively (Figure 1) [29,36]. These flavonoids, isoflavonoids and neoflavonoids, having three phenolic rings as a basic skeleton, commonly utilizing a pyran ring to link two benzene rings, however, aurones, classified as the minor flavonoids, possess a furan ring instead of pyran ring, and chalcones, also classified in the minor group, have an open chain instead of a ring structure, such as pyran or furan ring, to conjugate the two aromatic rings (Figure 1) [28,29,30,31,32,33,34,36]. Additionally, the general flavonoids, having 2-phenylbenzopyran as a basic structure, are classified into six subgroups based on the structural variations of the pyran ring, and these subclasses are named as follows: (i) flavonols, (ii) flavanols, (iii) anthocyanidins, (iv) flavones, (v) flavanones, and (vi) flavanonols (Figure 1) [28,29,30,31,32,33,34,36].

### 4.1. Action Mechanism of Flavonoids against Oxidative Stress Based on Chemical Structure

The chemical structure of flavonoids enables a variety of substitutions on their backbone, resulting in the production of a variety of derivatives and contributing to exhibiting numerous biological activities, such as anti-oxidative, anti-allergenic, anti-viral, and anti-inflammatory actions [28,29,30,31,32,33,34,36]. These biological properties of flavonoids, which are derived from the structural feature that can easily be substituted with glycosides, methyl groups, hydroxyl group, and sulphates, contribute to protecting the plant against ultraviolet radiation, pathogens, insects, and herbivores by acting as phytoalexins, detoxifying agents, and anti-microbial compounds [28,29,30,31,32,33,34,36]. Most of the flavonoids are very potent antioxidants, which have often been associated with their health-promoting effects [29,36,38,116]. So, the usage of various pharmaceutical and nutritional effects of flavonoids observed in plants for the development of preventive and/or therapeutic strategies against lots of diseases, such as autoimmune disease, cardiovascular disease, cancer, and neurodegenerative disease, have been studied (Figure 1) [28,29,33,39,44,117,118,119].

Flavonoids, which belong to the plant phenolic compounds, are widely distributed in plants. These flavonoids have common structural skeleton known as flavan, and the general flavonoids are classified into 6 subgroups based on the structural variations of the pyran ring. The subclasses of flavonoids are named as follows: (i) flavanols, (ii) flavanones, (iii) flavanonols, (iv) flavones, (v) flavonols, and (vi) anthocyanidins. Flavonoids have a relatively low redox potential that can easily reduce free radicals in cellular respiration.

#### ROS Scavenging Activity of Flavonoids

**Figure 2:**
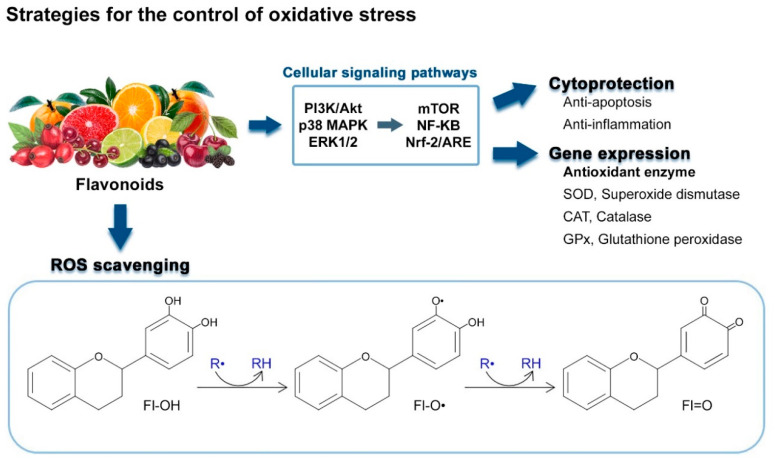
Mechanisms of flavonoids for the maintenance of redox balance.

According to a thermodynamic redox analysis based on the chemical feature of flavonoids, flavonoids (Fl-OH), which have low redox potentials, readily donate hydrogen (H) atoms to radicals, such as peroxyl, superoxide anion, and hydroxyl radical, resulting in the reduction of highly oxidized radicals. Flavonoids (Fl-O^•^) are converted into the aroxyl radical through a serial chemical reaction, and flavonoids are able to perform secondary radical scavenging activities by transferring the spare electron to the radicals to possess a stable potential themselves (Figure 2) [120,121]. Therefore, the capacity to act as antioxidants is reported in almost every compound of flavonoids:

Fl–OH + R^•^ → Fl–O^•^ + RH (9)

Fl–O^•^ + R^•^ → Fl = O + RH (10)

The redox potentials of flavonoids are determined depending upon the structural changes of the basic skeleton in the chemical configuration, substitution, and hydroxylated levels [120,121]. Particularly, the rate of hydroxylation is the key determinant of the anti-oxidative bioactivity of flavonoids, such as radical scavenging and metal ion chelation [36,41,122]. Hydroxyl groups of flavonoids, which are generally observed in positions 3, 5, 7, 2, 3′, 4′, and 5′ of flavonoids contribute to donating an H atom or receiving an electron and hydrogen from ROS and RNS to relatively stabilize themselves [36,41,122].

Flavonoids are representative antioxidants in nature. These natural compounds control the redox balance through (i) ROS scavenging activity-derived from their structural feature, (ii) cytoprotective effects, and (iii) regulation of genes expression encoding the antioxidant enzymes.

### 4.2. Anti-oxidative Effects of Flavonoids: Therapeutic Application of Flavonoids as Alternative Agents against PD

As previously mentioned, it has been well identified that a part of the PD pathogenesis is associated with oxidative stress induced by dopamine metabolism, mitochondrial dysfunction, and neurotoxic inflammation [57,116,123,124,125,126,127,128,129]. For these reasons, flavonoids, having potent free radical scavenging activity, have been considered and investigated as dietary supplements that prevent and suppress the PD pathogenesis. So, it has been studied whether the risk of PD pathogenesis can be decreased by a diet with flavonoids, such as fisetin, quercetin, naringin, baicalein, and genistein (Table 3) [31,41,122,130,131,132,133,134,135,136,137,138,139,140,141,142,143].

#### 4.2.1. Action Mechanisms of Flavonoids

It is well-defined that flavonoids can act as antioxidants by themselves, because they have ROS scavenging activity, which is derived from their chemical feature (Figure 2) [36,41,122]. Additionally, flavonoids can control the redox imbalance by regulating the antioxidant enzyme gene expression *via* Nrf2/ARE (nuclear erythroid 2-related factor 2/antioxidant response element) signaling pathway [144,145], and the activity of multiple cellular signaling pathways, such as Extracellular signal-regulated protein kinases 1 and 2 (ERK1/2) [42,146,147], c-Jun N-terminal kinases (JNK) [146,148], phosphatidylinositol 3-kinase/protein kinase B/mammalian target of rapamycin (PI3K/Akt/mTOR) [42,138,149], and mitogen-activated protein kinase (p38 MAPK) signaling pathway [148,150]. Moreover, flavonoids can suppress apoptosis and excessive inflammatory responses, thereby reducing the ROS that is incidentally generated by the activation of a cytotoxic mechanism (Figure 2) [43,138,151,152,153,154,155].

#### 4.2.2. Action Mechanisms of Flavonoids: Control of Mitochondrial Biogenesis and Oxidative Stress

The AMP-activated kinase (AMPK)/peroxisome proliferator-activated receptor-γ (PPARγ) coactivator-1α (PGC-1α) signaling pathway is one pathway that regulates mitochondrial biogenesis, and is also associated with the control of oxidative stress, which may increase the risk of onset of PD [156,157]. In fact, reports have indicated that the level of PGC-1α in the brains of patients with PD is decreased, compared with that of those who are non-diagnosed with PD [156]. PGC-1α is phosphorylated by AMPK, which is activated by physiological stimuli, such as exercise and starvation, and phosphorylated PGC-1α is translocated into the nucleus, and interacts with Nrf1 and Nrf2 to initiate gene expression for mitochondrial biogenesis [156]. Nrf1 and Nrf2 bind to the promoter regions of genes coding for ROS scavengers, fatty acid β-oxidation-related factors, OXPHOS, mitochondrial respiratory chain subunits, and mitochondrial transcription factor A (mtTFA) that drive the transcription and replication of mitochondrial DNA (mtDNA) [156]. In addition, the Nrf2 pathway can regulate mitophagy, which plays a pivotal role in the maintenance of mitochondrial homeostasis, contributing to the maintenance of neuronal viability and function. It is well understood that flavonoids such as genistein, (−)-epigallocatechin-3-O-gallate (EGCG), and quercetin can regulate PGC-1α activity through activation of Nrf2, resulting in direct/indirect regulation of Nrf1-dependent gene expression [156,157].

The Nrf2/ARE signaling pathway is the representative mechanism of flavonoids involved in the control of oxidative stress [144,145,158]. Nrf2 acts downstream of multiple cellular signaling pathways, which are associated with antioxidant pathway, heme/iron pathway, glutathione pathway, thioredoxin activity, iron/copper/zinc regulation, and intracellular protein accumulation regulation [158]. Flavonoids activate Nrf2 by phosphorylating *via* ERK1/2, JNK, PI3K/Akt/mTOR, and p38 MAPK signaling pathway [43,133,159,160,161]. Phosphorylated Nrf2 is dissociated with Kelch-like ECH-associated protein 1 (Keap1) and translocated into the nucleus inducing expression of anti-oxidative and anti-inflammatory factors [43,133]. Simultaneously, Keap1 inhibits the translocation of a nuclear factor kappa-light-chain-enhancer of activated B cells (NF-*Κ*B) from cytoplasm to nucleus, resulting in the downregulation of gene expression for pro-inflammatory cytokines, such as TNF-α and IL-1β [43,86,160]. Consequently, flavonoids can control the secondary oxidative stress through the induction of antioxidant enzymes and reduction of inflammation and cytotoxicity-induced ROS (Figure 2) [34,43,147]. Several studies evaluating the potential usage of flavonoids as preventive or therapeutic agents against PD by reducing the loss of dopaminergic neurons, vulnerable to oxidative stress, are organized in Table 3 [31,34,134,144,162,163,164,165,166,167,168,169].

Flavanols: Catechin and EGCG, abundantly present in green tea, can directly stabilize free radicals through the scavenging of ROS and chelation of metal ions. These compounds also regulate protein synthesis and cellular signaling pathways that are associated with the maintenance of redox balance involving compounds such as SOD, CAT, GSH, NADPH, and NF-ΚB. Moreover, reports suggest that long-term dietary supplementation with tea, which contains catechin and EGCG, delays the onset of PD (Table 3) [97,170,171].

Flavanones: Naringin, hesperetin, and pinocembrin are flavanones that have anti-oxidative effects and are beneficial agents for maintaining redox balance and promoting health. It is known that naringin, hesperetin, and pinocembrin attenuate neuronal death and inhibit mitochondrial dysfunction *via* the Nrf2/ARE signaling pathway in PD models in vivo and in vitro [31,45,137,138,145,164,172,173]. In addition, naringin induces gene expression associated with neuroprotection through mTORC1 activation (Table 3) [138].

Flavanonols: Ampelopsin, also known as dihydromyricetin, has a structure similar to myricetin. Ampelopsin protects neurons by increasing cellular antioxidant activities and anti-apoptotic activities through ERK1/2 and AKT signaling pathways. In addition, ampelopsin also attenuates 6-OHDA-induced neuronal death by regulating the acivity of the glycogen synthase kinase-3β (GSK-3β)/Nrf2/ARE signaling pathway [174,175]. Taxofilin exhibits a neuroprotective effect through its metal chealating properties (Table 3) [176,177].

Flavones: Chrysin, baicalein, apigenin, and luteolin are representative flavones. They suppress diverse neurotoxic events, such as neuroinflammation, abnormal apoptosis, and OS, through activation of ERK1/2, PI3K/Akt, and GSK-3β signaling pathways and upregulation of gene expression related to the anti-oxidative defense system (Table 3) [178,179,180,181,182,183,184].

Flavonols: Flavonols, including quercetin, myricetin, fisetin, rutin, and kaempferol, have been investigated as neuroprotective agents against neurodegenerative diseases such as AD and PD. They exhibit anti-oxidative and anti-inflammatory effects *via* cellular signaling pathways, such as ERK1/2, Akt, GSK-3β, and JNK, and it has been revealed that these flavonols can protect DA neurons in vivo and in vitro (Table 3) [45,122,132,133,185,186].

Anthocyanidins: Cyanidin, abundantly found in red berries, inhibits MPP^+^-induced mitochondrial dysfunction, and induces antioxidant-associated genes by regulating Nrf2-mediated gene expression. Pelargonidin alleviates 6-OHDA-induced neurotoxicity through the suppression of lipid oxidation in a rodent model of PD. Blueberry extracts, such as pelargonidin, peonidin, and petunidin, have multiple health-promoting properties, and it has been largely revealed that blueberries and pomegranate extract can protect neurons against neurotoxic events, such as apoptosis, inflammation, and OS (Table 3) [187,188,189].

Minor flavonoids: Genistein, an isoflavone abundantly found in soy bean, has been revealed to exert a protective mechanism through the restoration of decreased mitochondrial membrane potential in 6-OHDA-treated SK-N-SH neuroblastoma cells (Table 3) [180,190]. Phloretin (chalcones) can protect DA neuron and attenuate behavior deficits through inhibition of oxidative stress and neuroinflammation in an MPTP-induced mouse model of PD (Table 3) [191,192].

### 4.3. Other Beneficial Effects of Flavonoids as Potential Alternative Therapeutic Agents against PD

**Figure 3:**
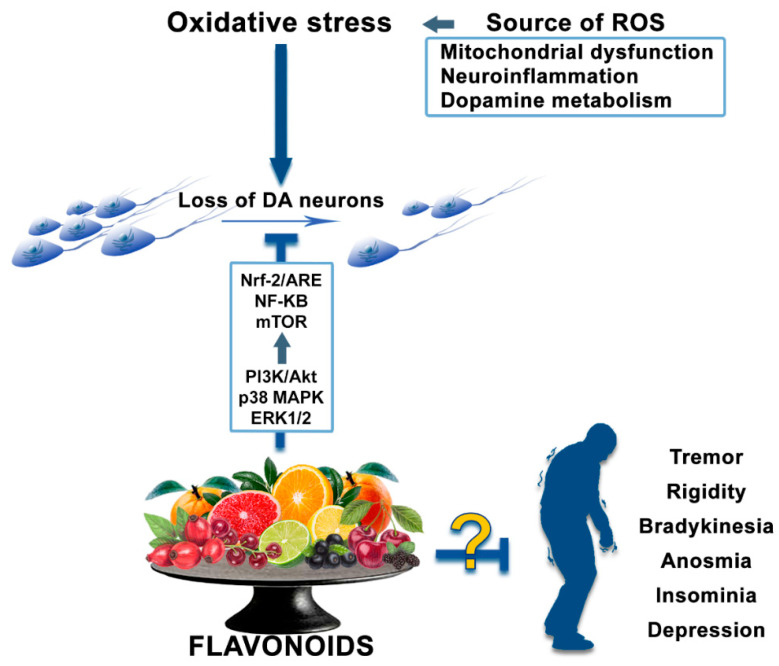
The potential of flavonoids as alternative therapeutic agents against Parkinson’s disease.

The representative bioactivity of flavonoids, plant-derived phenolic compounds, is an anti-oxidative effect. However, it has also been studied and revealed that flavonoids contribute to promoting the preservation of nigrostriatal DA pathway by providing other anti-parkinsonian effects, such as anti-apoptosis [193,194,195,196], anti-inflammation [136,138,146,153,197,198], and neurotrophic supports [136,138,199], and by inhibiting the α-syn fibrillation and oligomerization (Figure 3) [94,139,163,164,182,200,201].

The health-promoting bioactivities of flavonoids, including anti-diabetes, anti-tumor, anti-cell death, anti-inflammation, and anti-neurodegeneration, are well clarified. Particularly aspect of the neurodegeneration, flavonoids contribute to the control of excessive oxidative stress and inflammation, the clearance of misfolded proteins, and protection of neurons and their activities. In this review, we have organized PD, oxidative stress, and the potential of flavonoids as alternative therapeutic agents against PD.

#### 4.3.1. Anti-inflammatory Effects

The anti-inflammatory effect is well identified as a health-promoting property of flavonoids following the anti-oxidative effect. Several studies of in vivo and in vitro identified that flavonoids, such as naringin [136,138], silibinin [146,153], myricitrin [197], nobiletin [198], and astilbin [164], protect the DA neurons and/or DA axons against neurotoxin-induced neurodegeneration by suppressing the microglial activation and the subsequent release of pro-inflammatory cytokines, such as TNF-α and IL-1β [148,150,192,193]. Moreover, silibinin preserves the locomotor activity in the prothrombin kringle-2 (an endogenous microglial activator)-treated models, through the downregulation of ERK1/2 signaling pathway [146]. Daidzein and ampelopsin protect DA neurons against lipopolysaccharide (LPS, a bacterial-derived-exogenous microglial activator)-induced Neuroinflammation through the NF-*K*B and JAK2/STAT3 signaling pathways [174,175,202].

#### 4.3.2. Inhibitory Effects against α-Synuclein Oligomerization

Baicalein (5,6,7-trihydroxyflavone), which belongs to the flavones, has been found to demonstrate neuroprotective effects against PD models in vivo and in vitro [163,182]. According to previous studies, Baicalein exhibits neuroprotective effects in the nigrostriatal DA system against 6-OHDA, MPTP, and MPP^+^, and suppresses the accumulation of α-syn by inhibition of α-syn oligomer formation [163,183,203,204]. Apart from baicalein having a suppressive activity against α-syn fibrillation [205,206,207], apigenin [208,209], and EGCG [210,211] have also been found capable of preventing PD progression by inhibiting the aggregation and formation of α-syn oligomers [147,163,206,211].

#### 4.3.3. Induction of Neurotrophic Factors

Naringin (flavanone-7-O-glycoside) exhibits neuroprotective effects through anti-oxidation and anti-inflammation activity in a PD model [138,212]. Naringin is also able to induce neurotrophic factors to protect the nigrostriatal DA pathway through mTORC1 activation in the MPP^+^-treated animal models of PD [138]. It was confirmed that the neuroprotective effect of naringin is associated with GDNF inducing the diminishment of protective effect-derived from naringin by the treatment of GDNF neutralizing antibody [138]. Additionally, it is well established that the neuroprotective mechanism through the induction of neurotrophic factors by administration of flavonoids, such as, EGCG [213], fisetin [131], apigenin [181], luteolin [181], chrysin [199], and quercetin [185], in PD models in vivo and in vitro.

#### 4.3.4. Clinical Application of Flavonoids as an Alternative Therapy against PD

Green tea-derived flavonoids, such as catechin, epicatechin, and EGCG, have been widely studied to verify the beneficial effects of long-term supplementation of flavanols on aging-related disorders and neurodegenerative diseases. According to longitudinal studies, habitual consumption of tea, which abundantly contains catechin, epicatechin, and EGCG, is inversely correlated with the onset of PD [89,147,214,215]. A prospective study showed that intake of epicatechin and proanthocyanidin reduced the risk of PD pathogenesis through CREB-dependent transcriptional regulation, which is associated with neuronal viability, synaptic plasticity, and antioxidant enzyme activity [214]. In particularly, supplementation with blackcurrant, which contains proanthocyanidins, resulted in increased levels of cyclic glycine-proline (cGP), a metabolite of insulin-like growth factor-1 (IGF-1), in cerebrospinal fluid (CSF) of PD patients. The increase of cGP, a neuropeptide, suggest that supplementation with anthocyanidins can improve the function of IGF-1, resulting in the maintenance of neuronal function and viability [119]. In addition, proanthocyanidins may be able to increase dopamine concentration, inhibit monoamine oxidase-1 activity, and reduce DA neuronal loss in a 6-OHDA model [214,216,217].

Epidemiological studies have provided that dietary consumption of berries and herbs can reduce the risk of PD. According to Renoudet et al., basic diets supplemented with fisetin, found in berries, and hexacosanol-rich foods clinically improved most motor symptoms including cogwheel rigidity, bradykinesia, dystonia, and constricted arm swing with gait in a patient diagnosed with PD [186].

## 5. Conclusions

Oxidative stress is defined as a phenomenon resulting from a loss of balance between the production of ROS and the anti-oxidative defense system which is to detoxify and repair the cytotoxicity resulting from spare electrons and ROS [45,74]. Oxidative stress is frequently referred to as a risk factor of pathogenesis involved with a variety of diseases such as cardiovascular diseases, cancers, and neurodegenerative diseases [218]. Particularly in the brain, ROS production mainly occurs from catecholaminergic metabolism [22,23], mitochondrial dysfunction [14,19,24], and excessive neuroinflammation [3,8,9,25]. In addition, the DA neurons have striking vulnerability to oxidative stress-related neurotoxicity [14,20,21]. The maintenance of redox balance is a key factor for neuronal survival [219,220], therefore it is not surprising that any disruption in this balance induces neurodegeneration and neurological dysfunction in the nigrostriatal DA pathway, ultimately leading to the onset of PD.

Flavonoids are well known as natural antioxidants, also known to have numerous health-promoting bioactivities, such as anti-diabetes, anti-obesity, anti-cancer, and anti-neurodegeneration effects [39,117,130,221]. Over the last decades, the antioxidant effects of different types of flavonoids have been reported. Moreover, it has been studied whether flavonoids contribute to the neuroprotection and the improvement of locomotor activity through the anti-oxidative effect of them, due to the DA neurons having vulnerability for oxidative stress [179,221]. As mentioned above, flavonoids induce protective effects against neurodegeneration in models of PD in vivo and in vitro. In addition, flavonoids also protect the nigrostriatal DA system through the suppression of α-syn aggregation [163,164,183,203,204,205,206,207,208,209,210,211], the induction of neurotrophic factors [131,138,181,185,199,212,213], and inhibition of neurotoxic inflammation [138,146,147,148,150,153,164,197,198,199]. The previous studies show the possibility that a flavonoids-abundant diet can suppress the onset of PD in the population to whom are vulnerable to oxidative stress through the maintenance of redox balance, suggesting that the dietary supplement of flavonoids may induce health promotion against PD (Figure 3). Although there is no treatment for PD, levodopa, a dopamine precursor, is used for dopamine replacement therapy in PD. After administration, levodopa passes through the blood-brain barrier (BBB), it is metabolized into dopamine. Dopamine oxidation occurs by auto-oxidation and generates DAQ and ROS such as superoxide radicals [62]. Therefore, high concentrations of levodopa may induce excessive ROS production, leading to oxidative stress. Dietary supplementation with flavonoids and administration of levodopa may reduce this side effect by suppressing oxidative stress and provide a significant synergistic effect for PD treatment [222,223]. However, in order to apply the flavonoids as therapeutic agents against PD, it is necessary to clarify whether the flavonoids contribute to protection and restoration of neuronal function and neurite outgrowth in humans. Moreover, it is necessary to suggest that effective methods related to with dietary supplements of flavonoids including the concentration and the route of treatment. Although there are some problems, previous reports support the proposal that dietary supplement of flavonoids have the potential to be alternative agents for the prevention and treatment of PD.

## Figures and Tables

**Table 1:** Examples of reactive oxygen species.

Types of ROS.	
Oxygen Centered Radicals	Oxygen Centered Non-Radicals
Superoxide anion (O_2_^•−^) Hydroxyl radicals (^•^OH) Alkoxyl radicals (RO^•^) Peroxyl radicals (ROO^•^) Hydroperoxyl radicals (HO_2_^•^)	Hydrogen peroxide (H_2_O_2_) Hypochlorous acid (OCl^−^) Singlet oxygen (O_2_) Ozone (O_3_)

**Table 2:** A list of antioxidants and their chemical reactions.

Anti-oxidants		
Enzymatic Anti-oxidants		Non-enzymatic Anti-oxidants
Superoxide dismutase (SOD)	^•^O_2_^−^ + ^•^O_2_^−^ + 2H^+^ → H_2_O_2_ + O_2_	Glutathione (GSH)
Catalase	H_2_O_2_ + H_2_O_2_ → 2H_2_O + O_2_	Coenzyme Q (CoQ)
Glutathione peroxidase (GPx)	H_2_O_2_ + 2GSH → 2H_2_O + GSSG	Vitamine C & E

**Table 3:** A list of flavonoids associated with Parkinson’s disease.

Subgroups	Backbone	Compounds
Flavanols	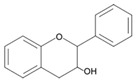	Catechin EGCG
Flavanones	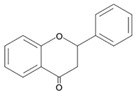	Naringin Hesperidin Pinocembrin Astilbin
Flavanonols	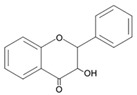	Ampelopsin Hesperetin Naringenin
Flavones	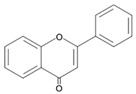	Chrysin Baicalein Apigenin Luteolin Tangeritin
Flavonols	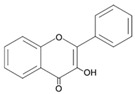	Quercetin Myricetin Kaempferol RutinFisetin
Anthocyanidins	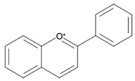	Cyanidin Pelargonidin Petunidin Malvidin
Isoflavones	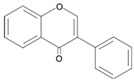	Genistein Daidzein Calycosin
Chalcones	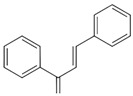	Phloretin Butein
